# Estrogen Rapidly Enhances Incisional Pain of Ovariectomized Rats Primarily through the G Protein-Coupled Estrogen Receptor

**DOI:** 10.3390/ijms150610479

**Published:** 2014-06-11

**Authors:** Guanghui An, Wenhui Li, Tao Yan, Shitong Li

**Affiliations:** 1 Department of Anesthesiology, Shanghai First People’s Hospital, Shanghai 200080, China; E-Mails: gh_An@163.com (G.A.); yantao74@gmail.com (T.Y.); 2 Department of Anesthesiology, Obstetrics and Gynecology Hospital of Fudan University, Shanghai 200090, China; E-Mail: panhongliang2000@gmail.com

**Keywords:** estrogen, estrogen receptors, G protein-coupled estrogen receptor (GPER), incisional pain

## Abstract

It has become increasingly apparent that the pain threshold of females and males varies in an estrogen dependent manner. To investigate the modulation of pain by estrogen and the molecular mechanisms involved in this process. A total of 48 rats were ovariectomized (OVX). At 14 and 20 days after OVX, rats were divided into eight groups: groups 1–4 were administered drugs intravenously (IV); groups 5–8 were administered through intrathecal (IT) catheter. Hind paw incision was made in all animals to determine incisional pain. Paw withdraw threshold (PWT) was tested prior to and 24 h after incision. The test drugs were applied 24 h after the incision. Rats were either IV or IT administered with: 17-β-estradiol (E2), G protein-coupled estrogen receptor (GPER)-selective agonist (G1), GPER-selective antagonist (G15) and E2 (G15 + E2), or solvent. Before and 30 min after IV drug administration and 20 min during the IT catheter administration, PWT was tested and recorded. 24 h after incisional surgery, the PWT of all rats significantly decreased. Both in the IV group and IT group: administration of E2 and G1 significantly decreased PWT. Neither administration of G15 + E2 nor solvent significantly changed PWT. Estrogen causes rapid reduction in the mechanical pain threshold of OVX rats via GPER.

## 1. Introduction

It has been shown that estrogen can influence pain threshold. In a study of healthy subjects, tested by a variety of stimuli including thermal stimulation, pressure stimulation and chemical stimulation, the pain threshold and tolerance were found to be lower in females than males [1,2,3,4]. In addition, females suffered more from clinical pain disorders than males in terms of migraines and trigeminal neuralgia [5].

In animals, the basal mechanical pain thresholds of male and female rats vary in an estrogen-dependent manner [6]. Administration of estrogen has been shown to result in sensitization of nociceptive neurons, resulting in a decrease of the pain threshold [7,8]. The paw withdraw threshold (PWT) significantly reduced after subcutaneous injection of estrogen into the paw. Subcutaneous injection of the selective agonist of the G protein-coupled estrogen receptor (GPER), GPER-selective agonist (G1) had similar results [9]. In the study by Evard *et al*. (2004), blockade of the endogenous synthesis of estrogens in quail markedly reduced the behavioral responsiveness to painful thermal stimulus within 1–5 min, and recovered within 30 min, indicating that estrogen had a rapid non-genomic effect on pain in the central nervous system (CNS) [10].

The results of studies investigating the effect of estrogen on pain often vary. The formalin pain model is commonly used as a model of chronic pain. Studies have shown that both estrogen replacement and administration of the estrogen receptor antagonist, tamoxifen, in the formalin model results in an anti-hyperalgesic effect [11,12]. In contrast, it has been reported that in a model of ovariectomized (OVX) rats with lower circulating levels of estrogen, the response to repetitive nociceptive stimulation was not altered compared to non-ovariectomized rats [13]. It has also been reported however, that OVX induces a hyperalgesic state of slow onset and long duration that can be reversed by estrogen [14].

Recently, the rapid effects of estrogen on nociceptive neurons have been reported. Studies have shown a reduction in PWT within a few minutes of a subcutaneous or intrathecal (IT) injection of estrogen [9,14]. Whilst several studies have reported that estrogen can induce a rapid increase in intracellular cAMP and Ca^2+^ concentration [1,15,16,17], the molecular mechanisms underlying the effects of estrogen remain unclear. In particular, few studies have investigated the role of the estrogen receptors in pain modulation. It is hypothesized that the reduction in pain threshold reported in conjunction with increased circulating estrogen may be modulated by estrogen receptors. The aim of the current study was to investigate the effects of estrogen in pain modulation and the possible molecular signaling pathways involved in this process.

A member of the novel protein kinase C family, PKCε, has been identified as an important intracellular mediator leading to the onset of mechanical hyperalgesia [18]. A study by Hucho *et al.* (2006) showed that estrogen controls PKCε-dependent mechanical hyperalgesia through direct action on nociceptive neurons [9]. Kuhn *et al*. reported similar results whereby GPER-selective agonist (G1) and ICI 182,780, a high affinity estrogen receptor antagonist, lead to translocation of PKCε in dorsal root ganglion (DRG) neurons to the plasma membrane [19]. Injection of G1 or ICI 182,780 into the hind paw of male rats rapidly induced PKCε-dependent mechanical hyperalgesia and PKCε-specific inhibitory peptide, εV1–2, could completely abolish the onset of estrogen-induced mechanical hyperalgesia [19]. These studies indicate that estrogen induces quick mechanical hyperalgesia through a PKCε-dependent pathway.

The *N*-methyl-d-aspartate (NMDA) receptor (NR) plays an important role in the initiation and maintenance of nociception. Both estrogen receptor α (ERα) and the NR1 subunit of the NMDA receptor are co-expressed in dorsal horn neurons, supporting a direct action of estradiol on NMDA receptors. The possible mechanisms include increasing the expression of NMDA receptors and changing the level of subunit phosphorylation [20]. The P2X (ligand-gated non-selective cation channel P2 purinoceptors ) receptors are expressed in DRG neurons. Studies have suggested that the P2X receptors in sensory neurons play a role in the generation and/or modulation of pain signaling from the periphery to the spinal cord [21]. Furthermore, it has been reported that estrogen inhibits P2X3 (ligand-gated non-selective cation channel P2 purinoceptors 3) receptor expression through a genomic mechanism [22] and the up-regulation of P2X3 receptors in DRG neurons from the OVX female rats may play a central role in mediating the abnormal nociceptive responses [23]. The mechanisms underlying the estrogen and P2X3 receptor-mediated nociceptive responses, however remain unclear.

## 2. Results and Discussion

### 2.1. Hind-Paw Withdrawal Threshold (PWT)

**Figure 1:**
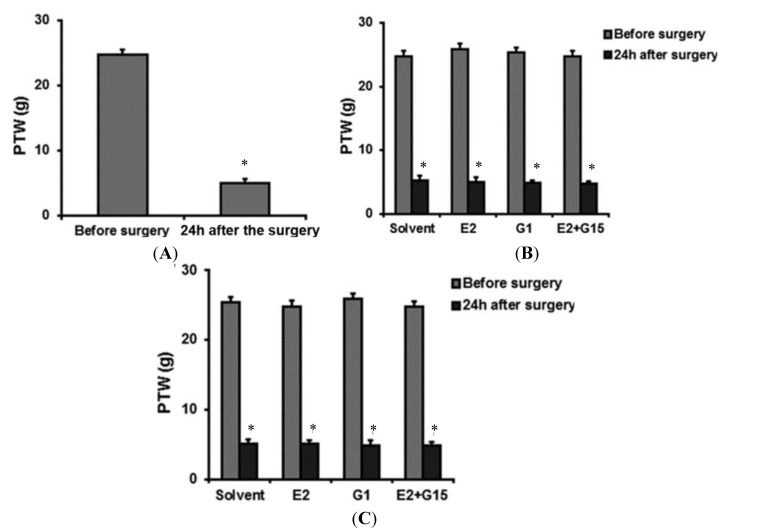
(**A**) The PWT of rats before and 24 h after the incisional surgery. The PWT of the rats dropped significantly 24 h after the incisional surgery * *p* < 0.05; (**B**) The PWT of rats before and after the incisional surgery of the intravenously (IV) group before administration of the indicated drug/drugs [17-β-estradiol (E2), GPER-selective agonist (G1), E2 + GPER-selective antagonist (G15)]. The PWT is presented as mean ± standard error of the mean (SEM). There was no statistically significant difference between the groups; and (**C**) The PWT of rats before and after the incisional surgery of the intrathecal (IT) group before administration of the indicated drug/drugs (E2, G1, E2 + G15). The PWT was presented as mean ± SEM in the figure. There was no statistically significant difference between the groups.

There was no significant difference in PWT between the groups before the incision surgery, and the PWT dropped significantly 24 h after the surgery (Table 1, Figure 1A–C). Still there were no significant differences between each groups 24 h after the surgery. The data of each group are shown in Table 1, Figure 1A–C.

**Table 1:** The paw withdrawal threshold (PWT) of all the groups, before surgery, before drugs and after drugs.

Administration	Group	Before Surgery	Before Drugs	After Drugs
IV	Solvent	24.8 ± 0.78 g	5.23 ± 0.72 g	5.00 ± 0.71 g
	E2	25.9 ± 0.88 g	5.03 ± 0.74 g	2.65 ± 0.66 g
	G1	25.4 ± 0.78 g	4.87 ± 0.40 g	2.50 ± 0.58 g
	E2 + G15	24.8 ± 0.78 g	4.67 ± 0.40 g	4.60 ± 0.51 g
IT	Solvent	25.4 ± 0.78 g	5.10 ± 0.63 g	5.05 ± 0.61 g
	E2	24.8 ± 0.88 g	5.10 ± 0.56 g	1.98 ± 0.36 g
	G1	25.9 ± 0.78 g	4.80 ± 0.86 g	2.56 ± 0.33 g
	E2 + G15	24.8 ± 0.78 g	4.90 ± 0.41 g	4.48 ± 0.55 g
Total		25.2 ± 0.49 g	4.90 ± 0.20 g	

### 2.2. Intravenously (IV) Group

#### 2.2.1. The Effect of 17-β-Estradiol (E2) Administration on PWT

**Figure 2:**
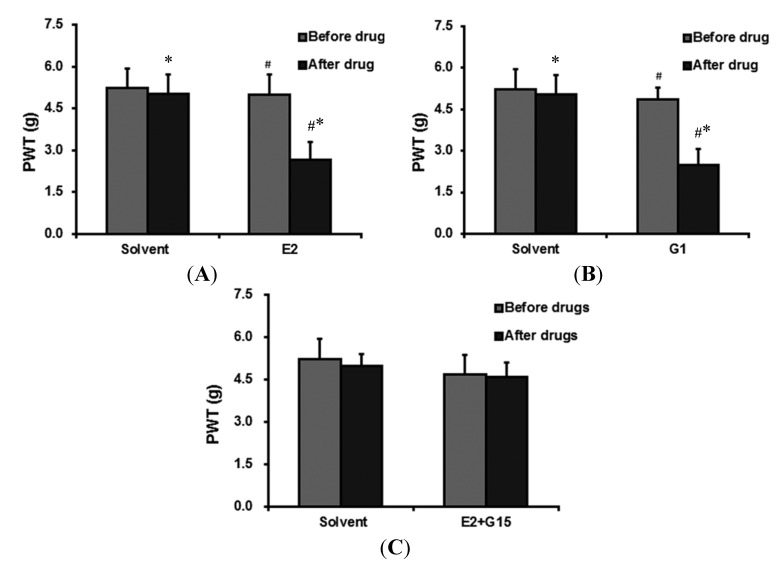
(**A**) The PWT of rats before and 30 min after the administration of solvent and E2 through the caudal vein. The PWT dropped significantly 30 min after administration of E2. * *p* < 0.05, compared with PWT before the administration; # *p* < 0.05, compared with the PWT of the solvent group after the drug administration; (**B**) The PWT of rats before and 30 min after the administration of solvent and G1 through the caudal vein. The PWT dropped significantly 30 min after G protein-coupled estrogen receptor (GPER)-selective agonist (G1) administration. * *p* < 0.05, compared with the PWT before the administration; # *p* < 0.05, compared with the PWT of the solvent group after the drug administration; and (**C**) The PWT of rats before and 30 min after the administration of solvent and G15 + E2 through the caudal vein. The PWT of the G15 + E2 group decreased but this was not statistically significant.

Twenty-four hours after incisional surgery, a high dose of E2 was administered to the OVX rats through the caudal vein. The results showed a significant decrease in the PWT of the incisioned hind-paw within 30 min after the administration of the E2 compared with the solvent group (Table 1 and Figure 2A).

#### 2.2.2. G Protein-Coupled Estrogen Receptor (GPER)-Selective Agonist (G1) Administration Ecreases PWT

In order to investigate the hypothesis that GPER was involved in the rapid action of estrogen, the GPER-selective agonist G1 was administered. A single dose of G1 (3 μg) was administered to OVX rats in the same way as E2. There was a significant difference between the PWTs of the pre-injection and post-injection group (4.87 ± 0.40 and 2.50 ± 0.58 g, respectively; Figure 2B, *p* < 0.05; *n* = 6).

#### 2.2.3. The Effect of 17-β-Estradiol (E2) + GPER-Selective Antagonist (G15) Administration on PWT

To substantiate the finding that the estrogen receptor (ER) GPER mediates the above rapid effect of estrogen on pain modulation, whether G15, a GPER-selective antagonist, could block the effect of E2 was investigated. Three minutes after the administration of E2, a single dose of G15 (E2:G15 = 1:7.4) was administered to the rats via the caudal vein. There was no significant difference between before drug administration and 30 min after the administration of E2 + G15 (Table 1 and Figure 2C).

### 2.3. Intrathecal (IT) Group

#### 2.3.1. The Effect of E2 Administration on PWT

**Figure 3:**
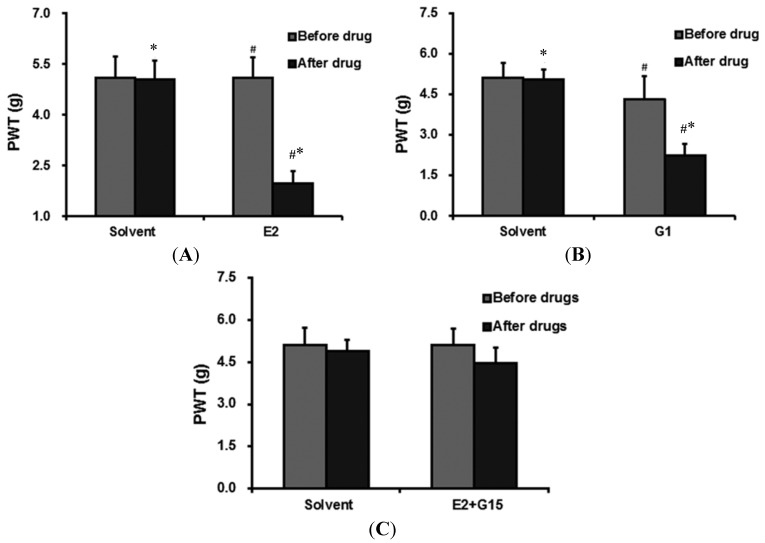
(**A**) The PWT of rats before and 20 min after the administration of solvent and E2 through intrathecal catheter. The PWT dropped significantly 30min after E2 adminisctration. * *p* < 0.05, compared with the PWT before the administration; # *p* < 0.05, compared with the PWT of the solvent group after drug administration; (**B**) The PWT of rats before and 20 min after the administration of solvent and G1 through intrathecal catheter. The PWT dropped significantly 30 min after applied with G1. * *p* < 0.05, compared with the PWT before the administration; # *p* < 0.05, compared with the PWT of the solvent group after the drug administration; and (**C**) The PWT of rats before and 30 min after the administration of solvent and G15 + E2 through the intrathecal catheter.

Twenty-four hours after incisional surgery, OVX rats were administered with E2 through the intrathecal catheter. The PWT around the wound decreased signficiantly from 5.10 ± 0.56 to 1.98 ± 0.36 g (*p* = 0.009) within 20 min. Administration of the solvent decreased the PWT from 5.10 ± 0.63 to 5.05 ± 0.61 g. The results showed that there was a significant decrease in the PWT of the incisioned hind-paw within 20 min after the administration of the E2 compared with the solvent group (Figure 3A).

#### 2.3.2. G1 Administration Decreases PWT

Twenty-four hours after the incisional surgery, OVX rats were administered G1 through the intrathecal catheter. The PWT around the wound decreased significantly from 4.30± 0.88 to 2.25 ± 0.42 g within 20 min (Table 1 and Figure 3B).

#### 2.3.3. The Effect of E2 + G15 Administration on PWT

Twenty-four hours after incisional surgery, OVX rats were administered with G15 + E2 through the intrathecal catheter. The PWT around the wound decreased, however this decrease was not significant. There was no significant difference between the E2 + G15 group and the solvent group (Table 1 and Figure 3C).

### 2.4. Discussion

The present study aimed to identify the molecular signaling pathways involved in the estrogen-related regulation of pain. Previous studies have shown that estrogen can influence pain behavior in both humans and animals [19]. The PWT in animals is significantly reduced following subcutaneous injection of estrogen into the animal paw [9]. In accordance with previous research, the present study showed that administration of estrogen induced rapid mechanical hyperalgesia in OVX rats following incisional surgery.

The pathological effects of estrogen are mediated by several molecular signaling pathways, the primary pathway being activation of the estrogen receptors, namely ERα and ERβ. These receptors are primarily located in the nucleus, belong to the steroid hormone receptor super-family and function as ligand-activated transcription factors. Estrogen regulates transcription through the ERs in two ways: (i) the classic activation whereby estrogen binds to the ERs, inducing transcription; and (ii) via the activator protein 1 (AP-1) enhancer elements pathway. The effect of estrogen via activation of the ERs by these pathways can often take hours if not days to be observed [24]. Interestingly, the present study reports a novel molecular signaling pathway of estrogen-related regulation of pain.

The G protein coupled estrogen receptors, GPERs, another form of estrogen receptors, are primarily located in the cell membrane. Estrogen can bind to GPERs acting rapidly through a nongenetic pathway. The GPERs were first isolated from B cells in 1996 by Carmeci *et al.* [25], and named GPR30 in 1997. The International Union of Pharmacology then officially named the receptor GPER. As a member of the G protein coupled receptor (GPCR) family, GPER has a 7-transmembrane hydrophobic domain, which consists of 20–26 amino acids interconnected by six alternating extracellular and intracellular loops [26]. Similar to GPCRs, GPER has an exceptionally conserved sequence, the Asp–Arg–Tyr triplet (DRY). DRY is located in the second intra-cytoplasmic loop after the third transmembrane domain, and is believed to play a role in signal transduction [27]. Several studies have shown that E2 can induce a rapid increase (within minutes and even seconds) of intracellular cAMP and Ca^2+^ concentration through GPER [1,15,16,17]. It has been reported both *in vitro* and *in vivo* that E2 can act rapidly, and it has been speculated that the receptor involved in this rapid effect of E2 was GPER [7,9,19]. Consequently, in the present study, the GPER’s selective agonists G1 and antagonist G15 were investigated.

The GPER-specific compound 1, G1, a substituted tetrahydro-3*H*-cyclopenta[c]-quinoline, does neither bind nor activate the classical nuclear receptors, but rather binds to GPER with high affinity and selectivity [28]. A G1 analog, G15, binds to GPER with high affinity, however has no activating effects. G15 does not bind to either ERα or ERβ. It has been shown *in vivo* that G15 can block some of estrogen’s uterine and neurological responses and that GPER contributes to the effects initiated by estrogen [17]. The results of the current study are in agreement with these findings, since administration of G1 or G15 + E2 decreased the PWT. suggesting that estrogen rapidly enhanced the incisional pain of OVX rats primarily through the GPER.

GPER in the spinal cord may mediate the rapid effects of estrogen.

When E2 and G1 were administered intrathecally, the effects on PWT were more rapid and more significant than intravenous, indicating that E2 may act through activating the GPER in the spinal cord. There are many factors in pain progression, and each step can influence pain [14]. The unmyelinated C fibre, the cell body located in the dorsal root ganglia, primarily responds to mechanical, thermal and chemical stimulation [29]. In rats, *GPER* mRNA is expressed in the spinal cord, dorsal root ganglia, no dose ganglia, trigeminal ganglia, hippocampus, brain stem and hypothalam0us [30]. In the spinal cord, GPER is detected in the dorsal horn, particularly in the superficial layer [30,31]. Interestingly, aromatase (estrogen-synthase, which catalyzes the conversion of C19 androgens into estrogens) is also expressed specifically in the superficial layer of the dorsal horn [24].

To date, few studies have investigated the mechanisms for the regulation of GPER expression. It has been shown however, that GPER expression is down-regulated in DRG neurons of OVX female rats, and that the reduction in GPER expression can be recovered by estrogen replacement [31].

The current study consists of several limitations including the minimal evaluation of pain. A combination of pain evaluation methods would be beneficial, more accurate, and might further support the results. Only one dose of each drug was investigated. A test-dose response curve would add further insight into the findings of the study. Finally, further molecular biology investigations would provide a possible molecular mechanism for analgesic drugs.

## 3. Materials and Methods

### 3.1. Animals

Behavioral experiments were performed on female Sprague-Dawley (SD) rats (100–150 g). Animals were obtained from the Shanghai Experimental Animal Center (Chinese Academy of Sciences, Shanghai, China) and housed in a controlled environment in a 12 h light:12 h dark cycle. Food and water were available *ad libitum*. Female rats underwent ovariectomy (OVX) 2 days after being housed at the lab. The study was conducted in accordance with applicable guidelines for animal research and was approved by the Ethical and Research Committees of Shanghai First People’s Hospital (2009, No. 03).

### 3.2. Drugs

17-β-Estradiol (E2) was purchased from Sigma, Hong Kong, China. G1 and G15 were purchased from Tocris, Shanghai, China. All drugs were dissolved in sterilized phosphate buffered saline (PBS) solvent and 10% dimethyl sulfoxide.

### 3.3. Equipment

The VonFrey filaments were purchased from Stolting, Wood Dale, IL, USA (0.008–300 g). The intrathecal catheter [PE-10 (PE-0503)] tube, consisted of an outer diameter of 0.5 mm and an inner diameter of 0.25 mm (AniLab Software & Instruments Co., Ltd., Ningbo, China). The catheter was cut into 10 cm, a mark made at 7.5 cm and sterilized by ethylene oxide. The IT injection was operated using a 20 μL-micro-syringe (Shanghai Medical Instruments Co., Ltd., Shanghai, China).

### 3.4. Ovariectomized (OVX) Surgery

Rats were anesthetized by inhalation of 3% isoflurane with pure oxygen. A 2–3 cm longitudinal incision was made using an aseptic technique. The incision was then pulled laterally to open the peritoneum, white adipose tissue observed, the ovaries and fallopian tubes identified, and the ovaries removed. The skin was sutured and penicillin administered to prevent infection. The rats were housed separately following surgery.

### 3.5. The Incisional Pain Model

Between 14 and 20 days after ovariectomy, the incisional pain model was performed as previously described [32]. A 1 cm longitudinal incision was made through the skin, fascia and muscle of the plantar aspect of the left hind-paw under 2% isoflurane anesthesia. The skin was then sutured with two mattress sutures of 4-0 nylon. After surgery, the animals were housed individually.

### 3.6. Subarachnoid Catheter

Subarachnoid catheter placement was performed as previously described [32]. Rats were anesthetized by inhalation of 3% isoflurane with pure oxygen, and placed in a special device for intrathecal (IT) catheterization. The fur on the back of the rats was shaved, confirmation of the depth of anesthesia noted and breathing monitored. A 3 cm incision along the midpoints of the ears was made. Subcutaneous fat and superficial fascia were removed to expose the neck muscles. The neck muscles were isolated along the midline and the foramen magnum exposed. The dura mater was carefully pricked and the cerebrospinal fluid expelled. The PE-10 catheter was carefully placed into the subarachnoid space approximately 7.5 cm through the foramen magnum. Position of the catheter, sutured muscles and skin were confirmed. Indwelling 1–2 cm catheter in the neck was fixed to the skin of the neck, and burnt at the end of the catheter to close it. The rats recovered in individual cages after surgery. Three days after successful placement of the catheter, the surgical rats were ready for the incisional model.

### 3.7. Evaluation of the Incisional Pain Threshold

The mechanical threshold, determined as the hind-paw withdrawal threshold (PWT) was measured using von Frey filaments as described previously [33]. Briefly, rats were placed into individual containers and acclimatized to the test chambers for 15 min. A series of nine von Frey filaments (0.4, 0.7, 1.2, 2.0, 3.6, 5.5, 8.5, 15 and 26 g) were applied vertically to the plantar surface of the hind-paw for 7 s while the filament was bent. Positive responses included an abrupt withdrawal of the hind-paw from the stimulus or flinching behavior immediately following removal of the stimulus. In the absence of a response at a pressure of 26 g, a cut-off value was assigned to the animal. Tests were performed in duplicate with an approximate 3 min resting period between withdrawal responses. The estimate of mechanical pain threshold (EI50) was calculated by the Equation (1):

EI50 = *X*_f_ + *k* × *d* (1)

where *X*_f_ is the final test level, *d* is the log interval between intensities and *k* is the tabulated value for maximum likelihood estimate. The PWT of the two hind-paws of the rats were tested before the incisional surgery, 24 h afterward surgery and 30 min after the administration of drugs.

### 3.8. Administration of Drugs

Drug administration was performed 24 h after the incisional surgery in order to allow assessment of the action of analgesics on an established pain state and to remove the possibility that pain-relieving treatment during surgery or in the immediate postsurgical period could not alter the development of the behavioral hypersensitivity [34].

Twenty-four hours after the incisional operation, all the rats were coded, and randomly divided into two groups. In the intravenously (IV) group, the drugs were administered through the caudal vein of the rat, in the IT group drugs were administered through intrathecal catheter. Each group was divided into four subgroups: A, the E2 group was administered E2; B, the G1 group, was administered G1; C, the G15 group, was administered E2 + G15; and D, the control group, was administered the same volume of solvent. The researcher who performed the von Frey test was blind to the drugs given to the rats and the grouping of the rats.

### 3.9. Doses of the Drugs

IV group: The dose of E2 was 1000 ng, G1 3000 ng, G15 + E2 of 10,000 + 1000 ng with G15 administered 3 min after E2 administration. IT group: The dose of E2 was 10 ng, G1 10 ng, G15 + E2 of 10 + 10 ng with G15 and E2 premixed before administration [6,9,10,19,20,24].

### 3.10. Statistical Analysis

The main data presented are the mechanical thresholds (PWT) of the rats at different periods. All data are reported as mean ± standard error of the mean (SEM). All statistical comparisons were made with one-way analysis of variances (ANOVAs) followed by *post hoc* comparisons using SPSS 18.0, IBM, Armonk, NY, USA. A *p* value of *p* < 0.05 was considered statistically significant.

## 4. Conclusions

Estrogen can rapidly regulate the incisional pain of OVX rats, whether administered through the caudal vein or the intrathecal catheter. The regulation of pain via E2 is modulated through the G protein-coupled estrogen receptor, primarily, spinal cord GPER.
